# The Effects of Repeated Morphine Treatment on the Endogenous Cannabinoid System in the Ventral Tegmental Area

**DOI:** 10.3389/fphar.2021.632757

**Published:** 2021-04-16

**Authors:** Hong Zhang, Austin A. Lipinski, Erika Liktor-Busa, Angela F. Smith, Aubin Moutal, Rajesh Khanna, Paul R. Langlais, Tally M. Largent-Milnes, Todd W. Vanderah

**Affiliations:** ^1^ Department of Pharmacology, College of Medicine, University of Arizona, Tucson, AZ, United States; ^2^ Department of Medicine, Division of Endocrinology, College of Medicine, University of Arizona, Tucson, AZ, United States

**Keywords:** opioids, endocannabinnoid, cannabinoid receptor, endogenous cannabinoid system, ventral tegmental area, reward

## Abstract

The therapeutic utility of opioids is diminished by their ability to induce rewarding behaviors that may lead to opioid use disorder. Recently, the endogenous cannabinoid system has emerged as a hot topic in the study of opioid reward but relatively little is known about how repeated opioid exposure may affect the endogenous cannabinoid system in the mesolimbic reward circuitry. In the present study, we investigated how sustained morphine may modulate the endogenous cannabinoid system in the ventral tegmental area (VTA) of Sprague Dawley rats, a critical region in the mesolimbic reward circuitry. Studies here using proteomic analysis and quantitative real-time PCR (qRT-PCR) found that the VTA expresses 32 different proteins or genes related to the endogenous cannabinoid system; three of these proteins or genes (PLCγ2, ABHD6, and CB2R) were significantly affected after repeated morphine exposure (CB2R was only detected by qRT-PCR but not proteomics). We also identified that repeated morphine treatment does not alter either anandamide (AEA) or 2-arachidonoylglycerol (2-AG) levels in the VTA compared to saline treatment; however, there may be diminished levels of anandamide (AEA) production in the VTA 4 h after a single morphine injection in both chronic saline and morphine pretreated cohorts. Treating the animals with an inhibitor of 2-AG degradation significantly decreased repeated opioid rewarding behavior. Taken together, our studies reveal a potential influence of sustained opioids on the endocannabinoid system in the VTA, suggesting that the endogenous cannabinoid system may participate in the opioid-induced reward.

## Introduction

The opioid epidemic is a severe health problem in the United States. It is estimated that over 10 million Americans misused prescription opioids and approximate two million of them have use disorders (SAMHSA, 2019). This high prevalence of opioid misusers/abusers is coupled to a striking increase in emergency room visits and overdose deaths due to non-medical use of opioids (Kolodny et al., 2015). Recently, this situation is even getting worse due to the social isolation and overwhelming despair associated with the COVID-19 pandemic (Volkow, 2020). Despite these significant detriments associated with opioids, 50 million Americans who are suffering from pain still need opioids–as they remain the current most effective analgesics (Dahlhamer et al., 2018). This dilemma has now become a challenging question for the medical community and requires further research to suppress the addictive potential of opioids especially for long-term use.

Opioid-induced reward is an initial but critical step toward opioid abuse and addiction (Fields and Margolis, 2015). Therefore, exploring the underlying mechanisms of opioid reward is essential to develop novel therapies for the treatment of the opioid epidemic. The mesolimbic circuitry is the major component of the brain reward system (Volkow and Morales, 2015). This circuitry is comprised of dopaminergic neurons projecting from the ventral tegmental area (VTA) of the midbrain to the nucleus accumbens (NAc) in the ventral forebrain (Volkow and Morales, 2015). The activation of these dopaminergic neurons is thought to directly encode for a reward prediction signal by producing a rapid, phasic dopamine release in the NAc (Volkow and Morales, 2015), which, at least in part, mediates opioid-induced reward.

The endogenous cannabinoid system (ECS), formed by cannabinoid receptors, lipid-derived endogenous ligands, and enzymes that synthesize and degrade the endogenous ligands, has recently been implicated in the process of rewarding behavior formation mediated by the mesolimbic circuitry. As it was shown previously, the blockade of cannabinoid receptor 1 (CB1R) in the VTA or NAc significantly suppressed morphine-induced conditioned place preference (CPP) or heroin self-administration (Caillé and Parsons, 2006; Rashidy-Pour et al., 2013); while administration of a selective Cannabinoid receptor 2 (CB2R) agonist JWH015 effectively attenuated acute morphine-induced dopamine release in the NAc, as well as attenuated morphine-induced rewarding behavior when co-administered with morphine (Grenald et al., 2017). Additionally, systemic administration of an endocannabinoid 2-arachidonyl glycerol (2-AG) or the inhibitor of its degrading enzyme MAGL, JZL184, significantly enhanced dopamine release in NAc (Oleson et al., 2012; De Luca et al., 2014). Interestingly, another study utilizing a dual FAAH (the primary enzymes that hydrolyzes the endocannabinoid anandamide (AEA) in the brain)-MAGL inhibitor, SA-57, reduced heroin-reinforced nose poke behavior and the progressive ratio break point for heroin (Wilkerson et al., 2017). Although distinct phenomena might be observed from different studies, current evidence strongly suggests the ECS can modulate and possibly participate in the development of opioid reward.

Current data propose that the ECS modulates opioid reward primarily via a disinhibitory feedback loop mediated by the 2-AG/CB1R axis in the mesolimbic circuitry (Zlebnik and Cheer, 2016). During periods of burst firing, VTA dopamine neurons release 2-AG onto the presynaptic GABAergic terminals in VTA (Lecca et al., 2012). The CB1R activation by 2-AG disinhibits the GABA-mediated inhibition of dopamine neurons, thus enhancing dopamine neuron activity (Melis et al., 2004; Riegel and Lupica, 2004; Fitzgerald et al., 2012). Recently, studies showed that CB2Rs are functionally expressed on the VTA dopamine neurons and can significantly suppress the activity of these neurons in the presence of cocaine, suggesting a potential ECS-mediated modulatory mechanism of opioid reward (Zhang et al., 2014; Zhang et al., 2017).

Considering the major impact of the endocannabinoid system on the rewarding effects of opioids, one important question is how the ECS in the mesolimbic reward circuitry responds to sustained exposure of opioids. To date, only a few studies have explored this research question (Rubino et al., 1997; Cichewicz et al., 2001; Gonzalez et al., 2002; González et al., 2003; Viganò et al., 2003; Viganò et al., 2004; Caillé et al., 2007; Jin et al., 2014). However, none of these studies have investigated the possible changes in the VTA. Additionally, most of these studies merely focused on analyzing the expression of CB1Rs, which lack a comprehensive understanding of the alterations in the whole ECS. Therefore, our present study sought to 1) obtain a thorough picture of the ECS-related proteins expressed in the VTA and their possible alterations induced by repeated opioid treatment through unbiased quantitative proteomics and 2) investigate the VTA levels of the two major endocannabinoids (AEA and 2-AG) by *in vivo* microdialysis after repeated morphine treatment.

## Materials and Methods

### Animals

Two hundred and twenty-three male Sprague Dawley rats (7–8 weeks old), purchased from Envigo (Indianapolis, IN), were maintained in a climate-controlled room on a 12 h light-dark cycle and allowed food and water *ad libitum*. Rats were housed three per cage for all experiments except for those that received guide cannula implantation, which were housed individually. All procedures were approved by the University of Arizona Animal Care and Use Committee (Approval #06–110) and adhere to the guidelines issued by the National Institutes of Health and the International Association for the Study of Pain.

### Drug Treatment

MJN110, a selective MAGL inhibitor (MAGL IC_50_ = 9.1 nM, over 1,000-fold selectivity vs. FAAH (Niphakis et al., 2013)), was purchased from Cayman Chemical (#17583, Ann Arbor, MI). JWH015, a selective CB2R agonist (CB2R Ki = 13.8 nM, 28-fold selectivity vs. CB1R (Showalter et al., 1996)), was purchased from Tocris (#1341, Minneapolis, MN). MJN110 and JWH015 were dissolved in a vehicle solution consisting of 10% dimethyl sulfoxide, 10% Tween-80, and 80% saline for injection (1 ml/kg, i. p.) with dosages of 5 and 3 mg/kg, respectively, based on the previous studies (Niphakis et al., 2013; Grenald et al., 2017; Thompson et al., 2020). Morphine sulfate was obtained from the NIDA Drug Supply program and was dissolved in saline for injection (1 ml/kg). Sustained morphine administration as previously described was performed by intraperitoneal injections of morphine sulfate to rats at a dose of 5 mg/kg twice daily (9:00 am and 5:00 pm) for five consecutive days (Viganò et al., 2003; Tumati et al., 2012; Campbell et al., 2013), which was intended to mimic the repeated use and systemic administration of opioids in humans (Ballantyne and Mao, 2003; Abrams et al., 2011; Surratt et al., 2011; Volkow and McLellan, 2016). For conditioned place preference testing, rats received a total of five injections of morphine at a dose of 10 mg/kg intraperitoneally over five consecutive days.

### Ventral Tegmental Area Tissue Collection

One hour after the last morphine or saline injection, rats were anesthetized with ketamine (80 mg/kg)/xylazine (10 mg/kg) mix and transcardially perfused with phosphate buffered saline (pH 7.4). Brains were carefully removed, and the VTA tissues (bilateral) were rapidly dissected on ice by using disposable biopsy punches (1 mm diameter) according to the Paxinos and Watson Atlas (Paxinos and Watson, 2007). Immediately after tissue harvest, the VTA samples were snap frozen in liquid nitrogen and then stored at −80°C until they were used for proteomics, western blotting, and quantitative real-time polymerase chain reaction.

### Proteomics Analysis

#### In-gel Digestion

Each VTA sample (3–4 mg VTA tissue from one rat) was lyzed in 60 μL chilled RIPA buffer (#89900, Thermo Scientific, Rockford, IL) with protease inhibitor cocktail (1: 50 dilution, #B14002, Bimake, Houston, TX). Immediately after adding the lysis buffer, samples were homogenized via ultrasonication (3 short bursts) and centrifuged at 15,000 g for 10 min at 4°C. The supernatant was transferred into a clean 1.5 ml tube, and the protein concentration in the tissue lysates were determined using Pierce BCA protein assay kit (#23225, Thermo Scientific, Rockford, IL). The protein concentration of each sample was ∼2–3 μg/μL 100 μg boiled tissue lysate was separated by SDS-PAGE and stained with Bio-Safe Coomassie G-250 Stain (#1610786; Biorad, Hercules, CA). Each lane of the SDS-PAGE gel was cut into seven slices. The gel slices were subjected to trypsin digestion and the resulting peptides were purified by C^18^-based desalting exactly as previously described (Kruse et al., 2017; Parker et al., 2019).

#### Mass Spectrometry and Database Search

HPLC-ESI-MS/MS was performed in positive ion mode on a Thermo Scientific Orbitrap Fusion Lumos tribrid mass spectrometer fitted with an EASY-Spray Source (Thermo Scientific, San Jose, CA). NanoLC was performed as previously described (Kruse et al., 2017; Parker et al., 2019). Tandem mass spectra were extracted from Xcalibur “RAW” files and charge states were assigned using the ProteoWizard 2.1. x msConvert script using the default parameters. The fragment mass spectra were searched against the rattus SwissProt_2018 database (8,068 entries) using Mascot (Matrix Science, London, United Kingdom; version 2.4) using the default probability cut-off score. The search variables that were used were: 10 ppm mass tolerance for precursor ion masses and 0.5 Da for product ion masses; digestion with trypsin; a maximum of two missed tryptic cleavages; variable modifications of oxidation of methionine and phosphorylation of serine, threonine, and tyrosine. Cross-correlation of Mascot search results with X! Tandem was accomplished with Scaffold (version Scaffold_4.8.7; Proteome Software, Portland, OR, United States). Probability assessment of peptide assignments and protein identifications were made using Scaffold. Only peptides with ≥95% probability were considered.

#### Label-free Peptide/protein Quantification and Identification

Progenesis QI for proteomics software (version 2.4, Nonlinear Dynamics Ltd., Newcastle upon Tyne, United Kingdom) was used to perform ion-intensity based label-free quantification. In brief, in an automated format, raw files were imported and converted into two-dimensional maps (*y*-axis = time, *x*-axis = *m*/z) followed by selection of a reference run for alignment purposes. An aggregate data set containing all peak information from all samples was created from the aligned runs, which was then further narrowed down by selecting only +2, +3, and +4 charged ions for further analysis. The samples were then grouped and a peak list of fragment ion spectra from only the top eight most intense precursors of a feature was exported in Mascot generic file (.mgf) format and searched against the rattus SwissProt_2018 database (8,068 entries) using Mascot (Matrix Science, London, United Kingdom; version 2.4). The search variables that were used were: 10 ppm mass tolerance for precursor ion masses and 0.5 Da for product ion masses; digestion with trypsin; a maximum of two missed tryptic cleavages; variable modifications of oxidation of methionine and phosphorylation of serine, threonine, and tyrosine; 13C = 1. The resulting Mascot. xml file was then imported into Progenesis, allowing for peptide/protein assignment, while peptides with a Mascot Ion Score of <25 were not considered for further analysis. Protein quantification was performed using only non-conflicting peptides and precursor ion-abundance values were normalized in a run to those in a reference run (not necessarily the same as the alignment reference run). Principal component analysis and unbiased hierarchal clustering analysis (heat map) was performed in Perseus (Tyanova et al., 2016; Tyanova and Cox, 2018) while Volcano plots were generated in Rstudio. Gene ontology (GO) and Kyoto Encyclopedia of Genes and Genomes (KEGG) pathway enrichment analysis was performed with DAVID (Huang et al., 2009).

### Western Blotting

Tissue lysates were prepared as for the proteomics analysis (methods above). Protein samples from each tissue lysate were resolved on 10% SDS-polyacrylamide gels (Criterion TGX, Bio-rad, Hercules, CA) and subsequently transferred to polyvinylidene difluoride (PVDF) membranes (Bio-rad, Hercules, CA). PVDF Membrane was blocked with 5% BSA in Tris-buffered saline containing 0.5% (v/v) Tween-20 (TBST) for 1 h at room temperature, and then incubated with different primary antibodies, including anti-rat CB1R antibody (rabbit polyclonal, 1:1,000 dilution), anti-rat DAGLα antibody (rabbit polyclonal, 1:4,000 dilution), anti-rat MAGL antibody (rabbit polyclonal, 1:2,000 dilution), and anti-α-tubulin (cp06, Calbiochem; mouse monoclonal, 1:50,000 dilution). All antibodies were diluted in Tris-buffered saline containing 0.5% (v/v) Tween-20 (TBST) and 3% (w/v) bovine serum albumin (BSA). Anti-rat CB1, DAGLα, and MAGL antibodies are kind gifts from Dr Ken Mackie. HRP-linked anti-rabbit IgG (7,074, Cell Signaling, Danvers, MA) and HRP-linked anti-mouse IgG (7,076, Cell Signaling, Danvers, MA) were used as the secondary antibodies for corresponding primary antibodies. The membranes were developed by using Clarity Western ECL substrate (#1705061, Bio-rad, Hercules, CA), and the blots were detected by GeneMate Blue Lite Autorad films (BioExpress, Kaysville, UT) and later quantitated with ImageJ 1.50i (Wayne Rasband, NIH, United States). All data were normalized to the α-tubulin signal for each sample. To probe a second protein on the same membranes, the membranes were washed in TBST and then stripped in the OneMinute Plus stripping buffer (GM6015, GM Biosciences, Frederick, MD). After a second run of wash in TBST, the membranes were blocked and then stained with another primary antibody.

### Quantitative Real-Time Polymerase Chain Reaction

Quantitative real-time polymerase chain reaction (qRT-PCR) was performed as described previously (Ibrahim et al., 2017). RNA was extracted from VTA tissues using TRIzol reagent (#15596026, Invitrogen, Carlsbad, CA) according to the manufacturer’s protocol. Briefly, samples were homogenized in 500 μL TRIzol reagent, and then 100 μL chloroform was added to each homogenate. After centrifugation, the upper aqueous phase containing RNA was transferred to a new tube and total RNA was subsequently precipitated after adding 250 μL isopropanol to the aqueous phase. Following by another centrifugation, the RNA precipitates were washed with 75% ethanol and resuspended in 20 μL DEPC-treated water (ThermoFisher, Grand Island, NY). cDNA was generated immediately after RNA extraction using the Maxima Reverse transcriptase kit (#K1641, ThermoFisher, Grand Island, NY) according to its manufacturer’s protocol. qRT-PCR analysis was performed using 5x HOT FIREPol EvaGreen qPCR Mix Plus (08–25–00001, Solis Biodyne, Estonia) on a CFX connect real-time PCR detection system (Bio-rad, Hercules, CA) according to the manufactures protocol. The relative mRNA expression for CB1R and CB2R genes was normalized to β-actin mRNA level and calculated with the ΔΔCt method (Livak and Schmittgen, 2001). The sequences of all specific primers were listed below: Rat CB1R (forward 5’- ACC​TAC​CTG​ATG​TTC​TGG​ATT​GGG -3’, reverse 5’- CGT​GTG​GAT​GAT​GAT​GCT​CTT​CTG -3′), Rat CB2R (forward 5’- CTC​GTA​CCT​GTT​CAT​CGG​CA -3’, reverse 5’- GTA​TCG​GTC​AAC​AGC​GGT​CA -3’) and Rat β-actin (forward TAA​GGC​CAA​CCG​TGA​AAA​GAT​GA -3’, reverse 5’- TAA​GGC​CAA​CCG​TGA​AAA​GAT​GA -3’). The primers for ABHD6 and PLCγ2 were acquired from Qiagen RT2 qPCR Primer Assays (ABHD6: PPR46100A-200 (NM_001007680); PLCγ2: PPR44457A-200 (NM_017168)).

### *In vivo* Endocannabinoid Analysis

#### Cannulation Implantation

Rats were anesthetized with ketamine (80 mg/kg)/xylazine (10 mg/kg) mix and secured in a stereotaxic apparatus (Stoelting, Wood Dale, IL). A unilateral microdialysis guide cannula (20 mm, MAB 2/6/9.20. G, SciPro, Sanborn, NY) was implanted into the ventral tegmental area (VTA) according to the Paxinos and Watson Atlas (Paxinos and Watson, 2007): AP −5.9 mm, ML +0.5 mm, and DV −8.2 mm from bregma. The guide cannula was fixed in place with skull screws and dental cement. All rats were injected with the antibiotic gentamicin (8 mg/kg, s. c.) to prevent infection. Morphine treatment started on the same day of surgery. Due to the small nuclei target, the successful rate of the cannula placement is ∼50%, with a total of *n* = 44 rats excluded for off target placement.

#### *In vivo* Microdialysis of Endocannabinoids in Awake Animals

*In vivo* Microdialysis experiments were conducted on the last day of repeated morphine treatment. The performance of microdialysis was modified from previous studies (Buczynski and Parsons, 2010; Wiskerke et al., 2012; Grenald et al., 2017). Two hours prior to sample collection, rats were lightly anesthetized for ∼2 min with 2% isoflurane for smooth insertion of a microdialysis probe (1 mm PES membrane and 15kD cut-off; MAB 6.20.1; 1 mm protruded beyond guide cannula) through and secured in the guide cannula. Immediately after probe insertion, the isoflurane anesthesia was discontinued and artificial cerebral spinal fluid (aCSF) containing 10% (w/v) hydroxypropyl-β-cyclodextrin (#16169, Cayman Chemical, Ann Arbor, MI) was perfused through the microdialysis probe and equilibrated within the brain tissue at a flow rate of 1 μL/min. Following this 2 h baseline period, all rats were injected with morphine (5 mg/kg, i. p.) and dialysate samples were collected every 30 min (30 μL total volume at each time point) for a total of 4 h. Collected samples were frozen in dry ice after each 30 min interval and subsequently stored at −80°C until further analysis. Upon completion, rats were sacrificed, and their brains were harvested and fixed in 10% formalin solution to verify cannula placement. Coronal slices containing the VTA were sectioned at a thickness of 40 μm on a cryostat. Guide cannula placements were verified visually and only those rats with correct cannula placement were used for final analysis.

#### Quantification of Endocannabinoid Contents in Microdialysates

Analysis of 2-AG and AEA contents in microdialysates was performed by the University of Arizona *Cancer* Center Analytical Chemistry Core on an Ultivo triple quadrupole mass spectrometer combined with a 1,290 Infinity II UPLC system (Agilent, Palo Alto, CA). Samples for analysis were prepared by mixing 10 μL internal standard solution (a mixture of 5.213 nM d_4_-AEA and 5.688 nM d_4_-2-AG in acetonitrile) to 20 μL microdialysate and then centrifuged at 15,800 xg for 5 min at 4°C. The supernatant was transferred to autosampler vials and 5 μL was injected for analysis. Chromatographic separation was achieved using an isocratic system of 21% 1 mM ammonium fluoride and 79% methanol on an Acquity UPLC BEH C-18 1.7u 2.1 × 100 mm column (Waters, Milford, MA) maintained at 60°C with a flow rate of 400 μL/min. After each injection the column was washed with 90% methanol for 1 min and then re-equilibrated for 5 min prior to the next injection. The mass spectrometer was operated in electrospray positive mode with a gas temperature of 150°C at a flow of 5 L/min, nebulizer at 15 psi, capillary voltage of 4,500 V, sheath gas at 400°C with a flow of 12 L/min and nozzle voltage of 300 V. The transitions monitored were: *m/z*348.3→*m/z*287.3 (d_0_-AEA), *m/z*352.3→*m/z*287.4 (d_4_-AEA), *m/z*379.3→*m/z*287.2 and 269.2 (d_0_-2-AG), and *m/z*348.3→*m/z*287.2 and 296.1 (d_4_-2-AG). As 2-AG is reported to be relatively unstable and can rapidly convert to 1-AG (Caillé et al., 2007), the 2-AG and 1-AG peak areas were combined for all analyses in the present study. The quantification of AEA and 2-AG was achieved by using calibration curves, which were prepared by serial dilution of AEA and 2-AG stock solutions in 80% acetonitrile. The stock solutions of AEA, 2-AG, d_4_-AEA and d_4_-2-AG were purchased from Cayman Chemical (Ann Arbor, MI).

### Conditioned Place Preference

The procedure of conditioned place preference (CPP) was modified from our previous studies (Grenald et al., 2017; Sandweiss et al., 2018). Rats were preconditioned to a three-chambered CPP apparatus (San Diego Instruments, San Diego, CA) for 15 min to acquire their baseline preference for the two side chambers. Only rats that showed no basal preference (<80% of the total time) or aversion (>20% of the total time) to any end chambers were used and randomly assigned for further conditioning experiments over the next 5 days. In our experiments, approximate 10–20% rats were excluded every time due to basal preference. In the morning session of the first conditioning day, rats were pretreated with MJN110 (5 mg/kg, i. p.), JWH015 (3 mg/kg, i. p.) or vehicle, and then were returned to their home cages. To ensure that JWH015 is on board and interacting at CB2R, animals were injected with morphine (10 mg/kg, i. p.) or saline 30 min after the pretreatment. This treatment time point has been applied frequently by previous studies using JWH015 or similar compounds (Ma et al., 2012; Verty et al., 2015; Zhang et al., 2018). Immediately after morphine/saline treatment, animals were confined to one end chamber (drug-paired chamber) for 15 min. In the afternoon session, all rats were pretreated with vehicle followed by an injection of saline and paired with opposite end chamber (non-drug paired chamber) as a control. All chambers were thoroughly cleaned after each trial to prevent effects of scent on behavior in the following trials. The same procedures were repeated on the conditioning days 2–5 while the morning and afternoon sessions in days 2–4 were inverted to counter-balance a putative effect of the circadian rhythm. In the morning of the test day (day 6), rats could explore all chambers of the CPP box freely for 15 min and the total time they spent in each chamber was recorded to determine their chamber preference. The chamber preference is presented as CPP score, which is calculated as below:

CPP difference score = Time spent in drug‐paired chamber on the test day −Time spent in drug‐paired chamber on baseline day

### Statistical Analysis

Power analyses were performed on cumulated data using G*Power 3.1 software (Faul et al., 2009) to estimate the optimal numbers of animals required for each experiment, and we found the adequate statistical separation for each group to detect 0.80 between groups at *p* < 0.05. ANOVA was used to analyze the expression difference between repeated morphine and saline-treated samples in the proteomic analysis. Two-way ANOVA with Tukey’s and Sidak’s multiple comparisons tests were used to analyze the time effect and group difference of the endocannabinoid production, respectively. Unpaired *t* test was used to compare the expression difference in proteomic analysis, western blotting, qRT-PCR and MAGL activity assay. One-way ANOVA with Dunnett’s multiple comparisons test was used to analyze the CPP tests. All data are presented as mean ± standard error of the mean (SEM) and a value of *p* < 0.05 was accepted as statistically significant. GraphPad Prism 8.0 (Graph Pad Inc., San Diego, CA) was used to perform statistical analyses and generate plots.

## Results

### The Effects of Sustained Morphine on the Ventral Tegmental Area Proteome

**FIGURE 1:**
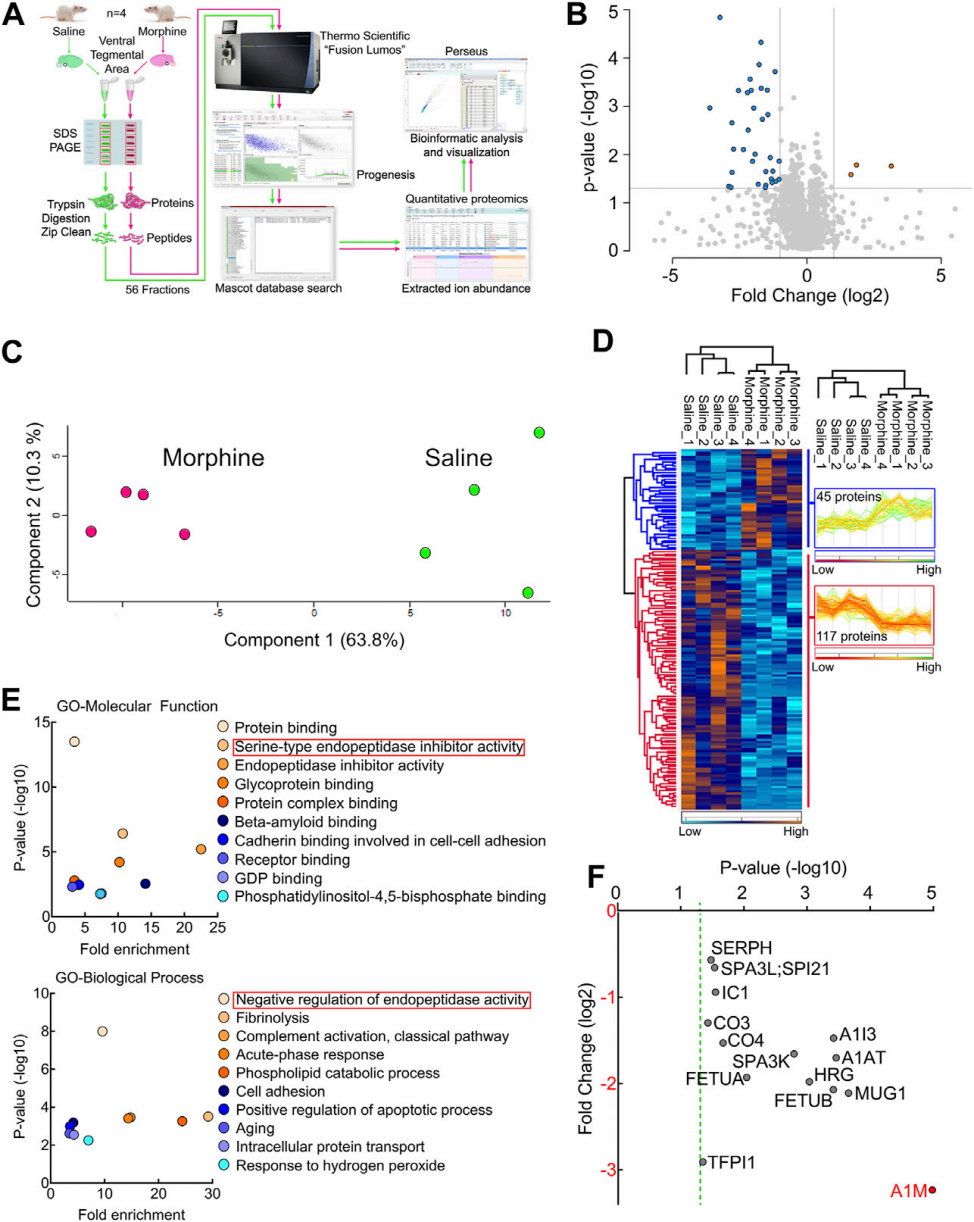
Proteomic analysis of the endogenous cannabinoid system-related proteins in rat VTA. **(A)** Experimental design for VTA tissue collection. **(B)** Schematic diagram of the label-free quantitative proteomics experimental approach. VTA tissues acquired from repeated morphine- or saline-treated rats were processed and used for performing proteomic analysis as described in Methods and Materials. **(C)** A volcano plot of the proteins identified in the VTA tissues treated with sustained morphine or saline. Above the horizontal gray line represents the cut-off for a *p* value of <0.05 while the two vertical lines represent the cut-off values of 2-fold change in either the positive or negative direction. **(D)** Unbiased principal component analysis (PCA) of the 162 significantly affected proteins from the 2-way ANOVA analysis of the quantitative proteomics data revealed that the protein expression differences of the individual biological samples within each group were consistent. **(E)** Unbiased hierarchical clustering of the 162 significantly affected proteins in the sustained morphine vs. saline treatment groups confirmed that the expression patterns across the different individual biological samples cluster together. **(F)** Scatter plots of the Gene Ontology (GO) enrichment findings for the significantly affected proteins after repeated morphine treatment. **(G)** Scatter plot of the endopeptidase inhibitors that are significantly affected by repeated morphine treatment. The Fold Change axis is labeled red to represent that treatment with morphine results in a decrease in the endopeptidase inhibitor proteins identified. The vertical dashed green line represents the *p* cut-off value of <0.05. α-1-macroglobulin is labeled in red to highlight the most significantly affected protein after morphine treatment. *n* = 4 per group.

To obtain a global picture of protein expression alterations in the VTA endogenous cannabinoid system after sustained opioid exposure, we carried out HPLC-ESI-MS/MS-based proteomic analysis of VTA tissues harvested from sustained morphine or saline-treated rats (Figure 1A). This analysis identified 3,680 total proteins across a total of 56 fractions from eight biological samples (Supplementary Table S1). 162 identified proteins were significantly (*p* < 0.05) affected by sustained morphine exposure, 37 of which had over 2-fold expression difference (Figure 1B; Supplementary Table S1). Of the 162 significantly regulated proteins, the expression levels of 117 were decreased after sustained morphine exposure and the levels of 45 were increased. Unbiased principal component analysis (PCA) of the 162 significantly affected proteins from the 2-way ANOVA analysis suggested a good consistency among the samples within each treatment group (Figure 1C), data that were supported by unbiased hierarchical clustering analysis (Figure 1D). The Gene Ontology (GO) enrichment and KEGG analyses of the significantly affected proteins were performed for GO-Molecular Function, Cellular Component and Biological Process as well as KEGG pathways, and the results are presented in Figure 1E; Supplementary Table S2. Interestingly, our analyses of GO-Molecular Function and GO-Biological Process suggest that the endopeptidase inhibitors are the most affected proteins in the VTA following repeated morphine treatment (All 14 proteins were reduced significantly and 11 of them had over 2-fold reduction in expression; Figures 1E, F; Supplementary Table S3). α-1-macroglobulin was the most significantly affected protein out of all 3,680 proteins, which has the largest fold change loss after morphine treatment of all the significantly affected proteins related to endopeptidase activity (labeled red in Figure 1F).

### Sustained Morphine Induces Proteomic Changes in the Ventral Tegmental Area Endogenous Cannabinoid System

**FIGURE 2:**
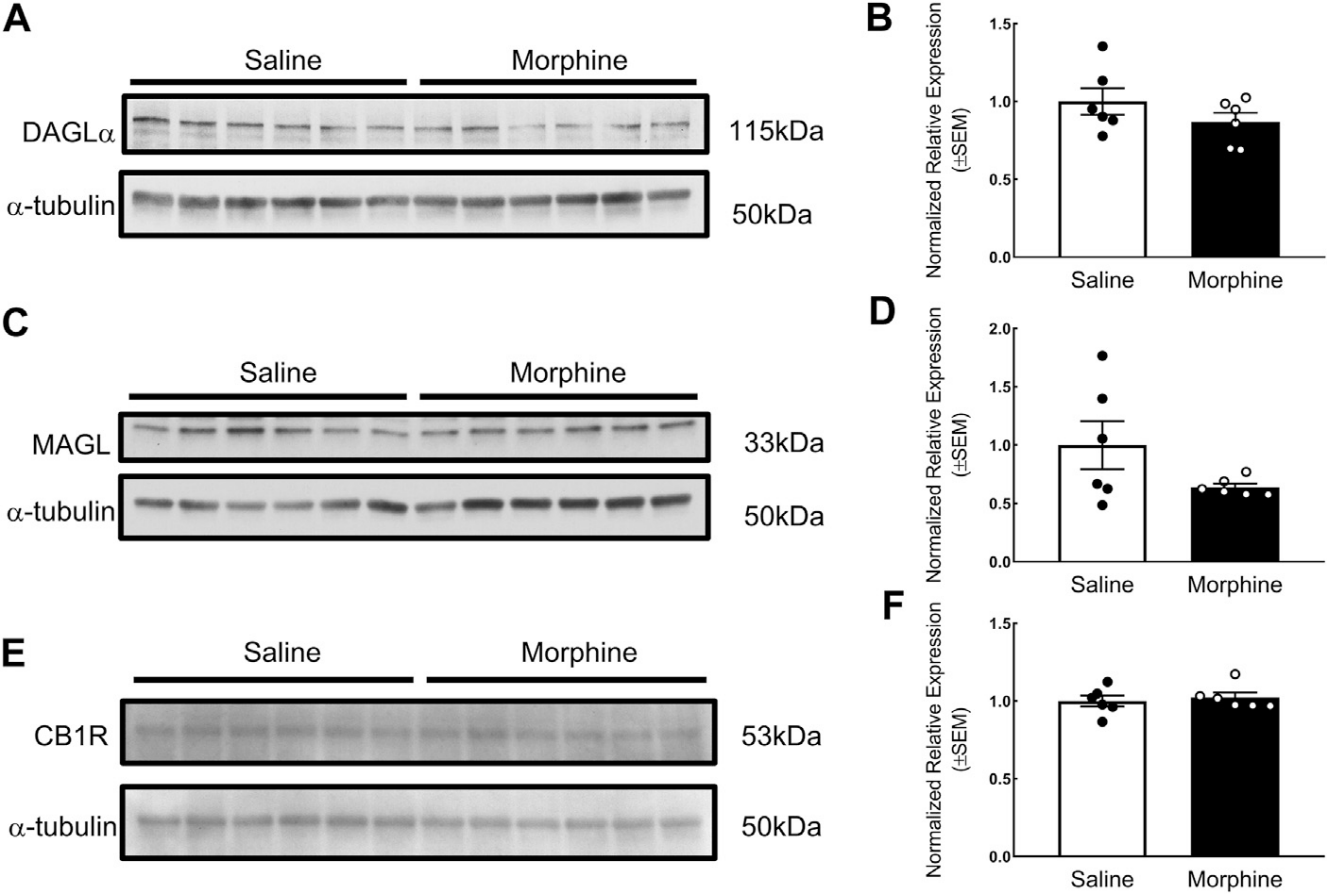
Verification of the effects of sustained morphine on DAGLα, MAGL and CB1R expression in the VTA. Rats were sacrificed after repeated morphine or saline treatment, and the VTA tissues were then collected and prepared for western blot analysis. **(A, C and E)** Samples were analyzed for the expression of DAGLα, MAGL, and CB1R. **(B, D and F)** Relative expression levels of DAGLα, MAGL, and CB1R were determined by densitometric analysis and normalized to *a*-tubulin (as internal control) in each lane. No significant difference in the expression of DAGLα, MAGL, and CB1R was observed between two treatment groups. Values represent the mean ± SEM, *n* = 6 per group.

**FIGURE 3:**
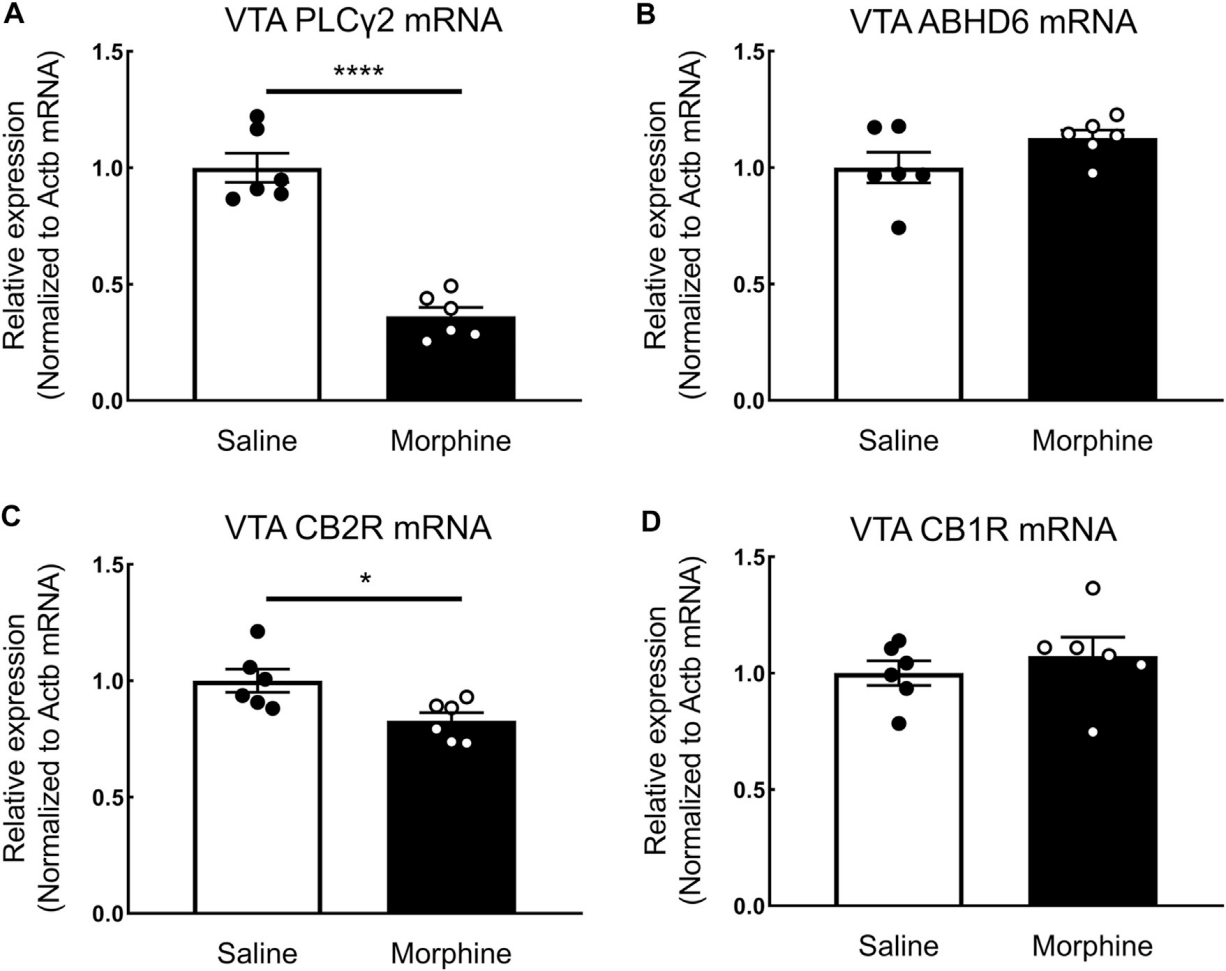
The effects of sustained morphine on the mRNA expression of PLCγ2, ABHD6, CB1Rs, and CB2Rs in the VTA. Rats were sacrificed after sustained morphine or saline treatment, and the VTA tissues were then collected and prepared for qRT-PCR analysis. Relative mRNA expression levels of **(A)** PLCγ2, **(B)** ABHD6, **(C)** CB2R, and **(D)** CB1R were determined by ΔΔCT method and normalized to β-actin mRNA level. PLCγ2 and CB2R mRNA expression was significantly decreased in repeated morphine treatment, but no significant difference in ABHD6 and CB1 expression was observed between two treatment groups. *p < 0.05, ****p < 0.0001, morphine vs. saline. Values represent the mean ± SEM, *n* = 6 per group.

In the proteomic analysis, 31 endogenous cannabinoid system-related proteins were detected in the VTA (Table 1). Two of these proteins, phospholipase Cγ-2 (PLCγ2) and α/β-hydrolase domain containing 6 (ABHD6), were significantly downregulated after sustained morphine exposure. To verify the results of our proteomic analysis, we examined the expression of PLCγ2 and ABHD6 as well as several proteins that are demonstrated as the major contributors to the endocannabinoid signaling in the central nervous system, including diacylglycerol lipase α (DAGLα), monoglyceride lipase (MAGL) and CB1R, using either western blotting or qPCR. Consistent with our proteomic data, DAGLα, MAGL and CB1R were not significantly altered in the VTA after repeated morphine treatment (DAGLα: t (10) = 1.27, *p* = 0.23; MAGL: t (10) = 1.73, *p* = 0.11; CB1R: t (10) = 0.47, *p* = 0.65) (Figures 2A–F), yet findings for the PLCγ2 using qPCR demonstrated PLCγ2 was significantly downregulated after morphine treatment (t (10) = 8.65, *p* < 0.0001) (Figure 3A). These results indicate the accuracy and reproducibility of the proteomic analysis (t (10) = 1.70, *p* = 0.12). Interestingly, ABHD6 detected by qPCR was not shown to be altered by sustained morphine (Figure 3B), suggesting its downregulation detected by proteomics may not be controlled at the transcription level.

**TABLE 1:** Expression alterations of the endogenous cannabinoid system-related proteins in the VTA after chronic morphine exposure.

Protein name	Gene name	MW (kDa)^a^	Fold change^b^	Anova P value^c^
Receptors for cannabinoids				
Cannabinoid receptor 1	CNR1	52.8	0.85	0.380
Transient receptor potential cation channel subfamily V member 1	TRPV1	94.9	0.54	0.924
Enzymes related to endocannabinoid synthesis				
1-Phosphatidylinositol 4,5-bisphosphate phosphodiesterase beta-1	PLCB1	138.3	1.04	0.595
1-Phosphatidylinositol 4,5-bisphosphate phosphodiesterase beta-3	PLCB3	139.4	0.88	0.164
1-Phosphatidylinositol 4,5-bisphosphate phosphodiesterase beta-4	PLCB4	134.4	1.04	0.721
1-Phosphatidylinositol 4,5-bisphosphate phosphodiesterase delta-1	PLCD1	85.9	1.01	0.971
1-Phosphatidylinositol 4,5-bisphosphate phosphodiesterase delta-4	PLCD4	88.9	0.64	0.059
1-Phosphatidylinositol 4,5-bisphosphate phosphodiesterase gamma-1	PLCG1	148.5	0.90	0.134
1-Phosphatidylinositol 4,5-bisphosphate phosphodiesterase gamma-2	PLCG2	147.6	0.63	0.035*
Glycerophosphodiester phosphodiesterase 1	GDE1	37.6	0.72	0.089
N-acyl-phosphatidylethanolamine-hydrolyzing phospholipase D	NAPEPLD	45.7	1.07	0.913
Phosphatidylinositol 3,4,5-trisphosphate 5-phosphatase 1	SHIP1	133.5	0.87	0.923
Sn1-specific diacylglycerol lipase alpha	DAGLA	115.2	0.53	0.107
Enzymes related to endocannabinoid degradation				
Arachidonate 12-lipoxygenase, 12 R type	ALOX12 B	80.7	0.92	0.354
Cytochrome P450 2C70	CYP2C70	56.1	1.07	0.365
Cytochrome P450 2D4	CYP2D4	56.6	1.04	0.833
Cytochrome P450 4F5	CYP4F5	60.6	0.72	0.105
Fatty-acid amide hydrolase 1	FAAH1	63.3	1.14	0.305
Monoacylglycerol lipase, abhydrolase domain containing 6	ABHD6	38.3	0.69	0.031*
Monoacylglycerol lipase, abhydrolase domain containing 12	ABHD12	45.3	1.03	0.648
Monoglyceride lipase	MGLL	33.5	1.00	0.956
N-acylethanolamine-hydrolyzing acid amidase	NAAA	40.3	0.81	0.410
Endocannabinoid transport proteins				
Fatty acid-binding protein 5	FABP5	15.1	0.85	0.495
Fatty acid-binding protein 7	FABP7	14.9	0.97	0.810
Heat shock 70 kDa protein 1 A	HSPA1A	70.1	1.59	0.143
Heat shock 70 kDa protein 1-like	HSPA1L	70.5	0.80	0.975
Heat shock-related 70 kDa protein 2	HSPA2	69.6	1.20	0.491
Heat shock 70 kDa protein 4	HSPA4	94.0	1.04	0.668
Heat shock 70 kDa protein 13	HSPA13	51.8	0.95	0.734
Heat shock 70 kDa protein 14	HSPA14	54.4	1.29	0.283
Regulatory protein				
CB1 cannabinoid receptor-interacting protein 1	CNRIP1	18.6	1.08	0.619

^a^ MW, molecular weight.

^b^ Expression difference is presented as the fold change of the protein abundance in the tissues after chronic morphine treatment: fold change = protein expression level (after sustained morphine treatment)/protein expression level (after saline treatment).

^c^ *p < 0.05.

CB2R, a critical member of the endogenous cannabinoid system, was recently demonstrated as playing a key role in drug addiction (Xi et al., 2011; Navarrete et al., 2013; Ortega-Álvaro et al., 2015; Grenald et al., 2017). However, due to its low expression level in the central nervous system, this receptor was not detected with current mass spectrometry-based or antibody-based techniques (Cécyre et al., 2014; Marchalant et al., 2014; Li and Kim, 2015). To examine the expression of CB2R in the VTA, we employed quantitative real-time PCR. Our results showed that sustained morphine significantly decreased the mRNA expression level of CB2Rs by 17% (t (10) = 2.82, *p* < 0.05) (Figure 3C). In contrast, no significant difference of CB1R expression between repeated morphine-treated and saline-treated groups was found (t (10) = 0.77, *p* = 0.46) (Figure 3D), which is consistent with our previous protein observation (Figure 2F).

### The Effects of Sustained Morphine on the Production of Endocannabinoids in the Ventral Tegmental Area

**FIGURE 4:**
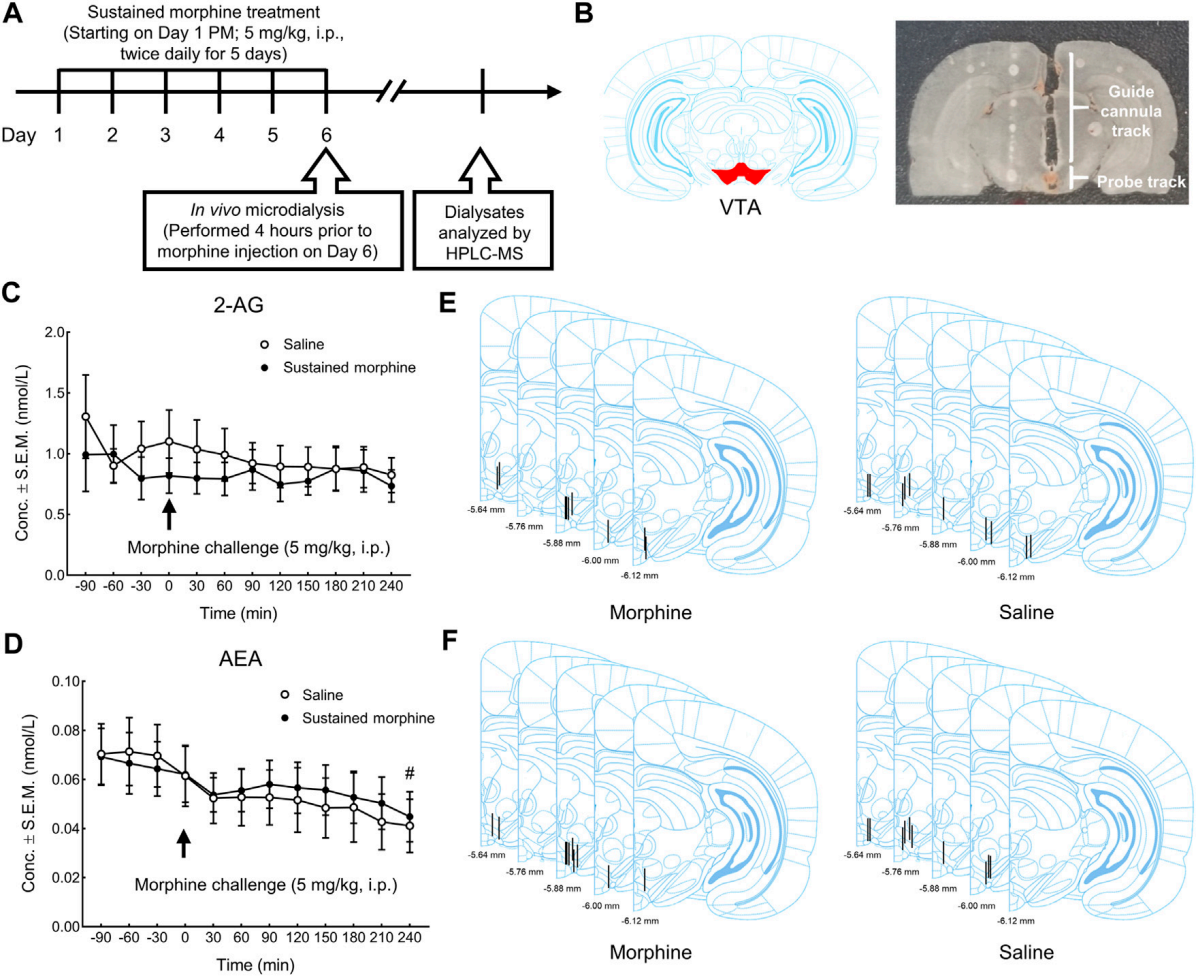
The effects of sustained morphine on the production of 2-AG and AEA in the VTA. *In vivo* microdialysis was performed on rats one day after repeated morphine treatment to determine the alterations of endocannabinoids in VTA. Microdialysis samples were collected every 30 min for a total of 6 h. After the first 2 h baseline (*t* = −120–0 min), all rats received an additional injection of morphine and the changes in the production of endocannabinoids was observed for the next 4 h (*t* = 0–240 min). **(A)** Experimental design for *in vivo* microdialysis of endocannabinoids. **(B)** Representative brain section of microdialysis guide cannula/probe implantation. **(C, D)** no significant difference in the production of either 2-AG or AEA was observed between treatment groups at baseline session or in the morphine challenge session. However, a significant time effect in AEA production was observed between the baseline period and the last time point of the morphine challenge session (t = 240 min). **(E, F)** Anatomical representatives of microdialysis guide cannula placements in VTA for the studies of 2-AG and AEA. ^#^ *p* < 0.05, *t* = 240 min vs. baseline (*t* = −90 to 0 min). Values represent the mean ± SEM, *n* = 9–10 per group.

Next, to determine the influence of sustained morphine on the production of the endocannabinoids 2-AG and AEA, we employed *in vivo* microdialysis in the VTA of awake rats after sustained morphine administration (Figures 4A,B). Our results found that, one day after repeated morphine treatment, the production of 2-AG in the VTA was not significantly changed compared to saline-treated group (Interaction: F (11, 187) = 0.72, *p* = 0.71; Time: F (2.714, 46.14) = 1.60, *p* = 0.21; Column factor: F (1, 17) = 0.32, *p* = 0.58; Subject: F (17, 187) = 31.89, *p* < 0.0001) (Figure 4C; t = −120–0 min). However, considering the possibility that sustained morphine-induced 2-AG alteration have returned to the baseline level, we performed an additional injection of morphine (5 mg/kg, i. p.) to both sustained morphine-treated and saline treated rats and determined whether the 2-AG production may be altered compared to the baseline levels. Again, no significant difference of 2-AG production was identified compared to baseline levels or between two treatment groups (Figure 4C).

In addition to 2-AG, we also investigated the effects of sustained morphine on AEA production. Similarly, no significant difference in AEA production was observed between repeated morphine-treated and saline treated rats before (baseline session; *t* = −120–0 min) or after the morphine challenge in both groups (morphine challenge session; *t* = 0–240 min) (Interaction: F (11, 198) = 0.41, *p* = 0.95; Time: F (2.045, 36.80) = 6.84, *p* < 0.01; Column factor: F (1, 18) = 0.02, *p* = 0.88; Subject: F (18, 198) = 59.01, *p* < 0.0001) (Figure 4D). Interestingly, we did identify a significant difference in AEA production between the baseline (*t* = −90 to 0 min) and the last time point of the morphine challenge session (*t* = 240 min) when data from both groups (chronic-saline and -morphine) were combined (*t* = −90 to 0 min vs. *t* = 240 min: *p* < 0.05) (Figure 4D), something not seen with 2-AG levels. The placement of all microdialysis guide cannulas was verified after experiments (Figures 4E,F).

### The Modulatory Effects of 2-AG and CB2R on Sustained Morphine-Induced Reward

**FIGURE 5:**
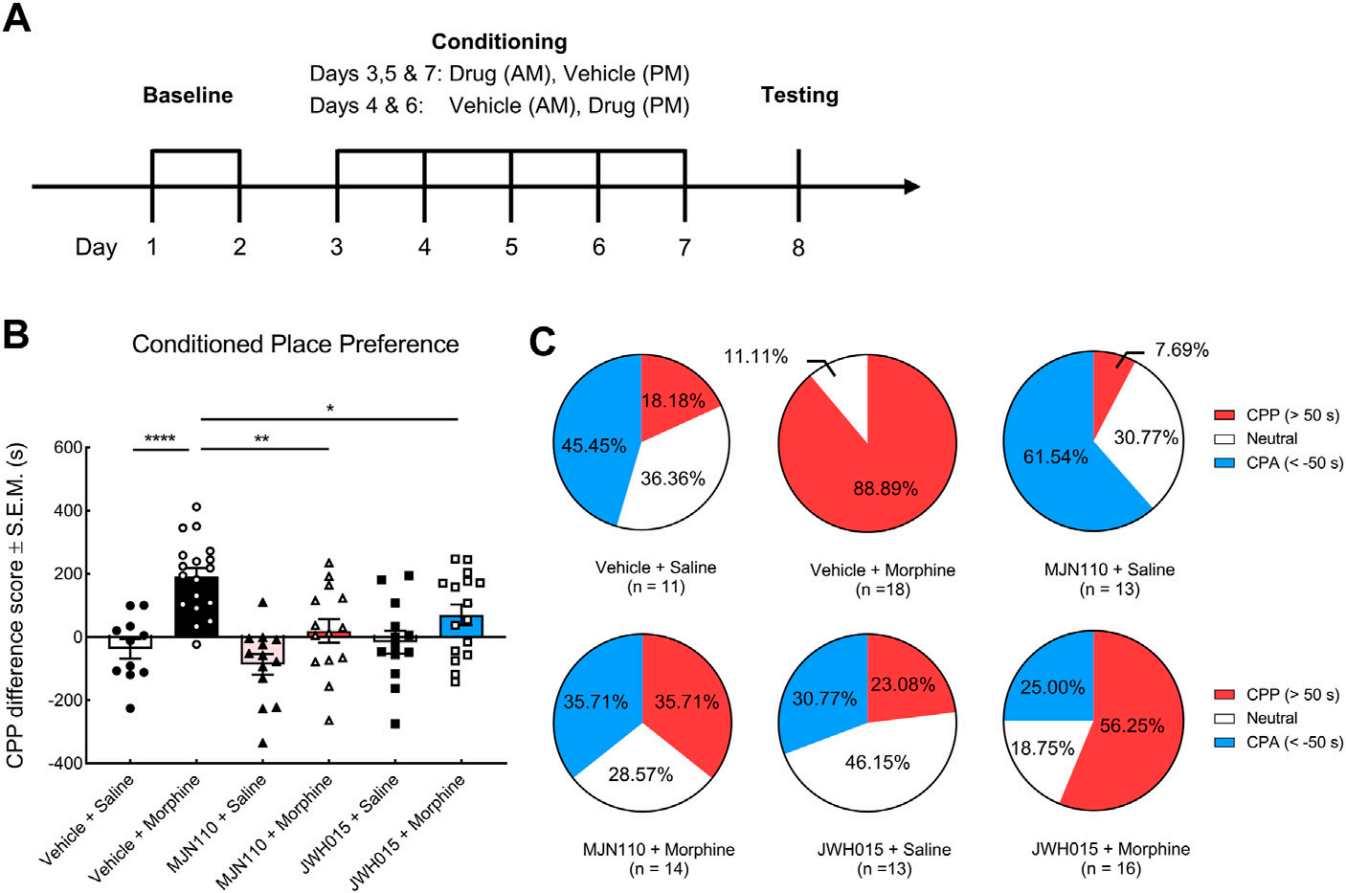
The effects of systemic MJN110 and JWH015 on the sustained morphine-induced conditioned place preference. Conditioned place preference was employed to investigate the modulatory effects of a selective MAGL inhibitor, MJN110, and a selective CB2R agonist, JWH015, on sustained morphine-induced reward. **(A)** Experimental design of conditioned place preference assay. After baseline testing to establish approximate equal times in the two end chambers, rats were paired with a treatment and one of the end chambers for 5°days. Their chamber preference (access to all chambers) was tested on day 8 with no treatment administered. **(B)** Rats that received sustained morphine presented a strong preference toward the drug-paired chamber but not saline-treated rats. MJN110 significantly suppressed morphine-induced preference. MJN110 may produce aversive effect on rats but no statistic difference was observed compared to the results from vehicle-saline-treated rats. JWH015 attenuated morphine-induced preference but did not alter the preference in saline-treated rats. **(C)** Pie plots showing the percentages of rats exhibiting conditioned place preference, aversion or no preference. CPP (red): Animals presenting remarked conditioned place preference (CPP difference score >50°s); CPA (blue): Animals presenting remarked conditioned place aversion (CPP difference score < −50°s); Neutral (white), Animals presenting no remarked preference or aversion (CPP difference score between −50 and 50°s). or *p < 0.05, **p < 0.01, ****p < 0.0001. Values represent the mean ± SEM, *n* = 11–18 per group.

Lastly, we briefly investigated the possible roles of the proteins and genes regulated by sustained morphine in the modulation of opioid reward hoping to identify potential target(s) for the treatment of opioid reward and addiction. According to the proteomic data, we found that the expression of PLCγ2 and ABHD6 was downregulated by repeated morphine treatment. These two proteins are possibly implicated in the production of 2-AG (Blankman et al., 2007; Kadamur and Ross, 2013). Although the *in vivo* microdialysis showed that sustained morphine does not modulate 2-AG level in VTA, it is still possible that 2-AG can exert regulatory effects on sustained morphine-induced reward. To examine this idea, we facilitated the production of endogenous 2-AG on sustained morphine-induced reward pharmacologically using conditioned place preference assay (Figure 5A). Our results showed that rats receiving sustained morphine exhibited a significant preference to the drug-paired chamber compared to the saline-treated rats reflecting by both CPP difference score and percentage of rats presenting CPP (F (5, 79) = 9.62, *p* < 0.0001; Vehicle + Morphine vs. Vehicle + Saline: *p* < 0.0001; CPP (>50°s): 88.89 vs. 18.18%) (Figures 5B,C). Increasing endogenous 2-AG tone by pretreatment with the selective MAGL inhibitor, MJN110 (5 mg/kg, i. p.), significantly reduced the time that animals spent in morphine-paired chambers and the proportion of animals showing CPP (Vehicle + Morphine vs. MJN110 + Morphine: *p* < 0.01; CPP (>50°s): 88.89 vs. 35.71%) (Figures 5B,C). Interestingly, we found that the rats received MJN110 alone showed a trend of aversion to drug-paired chamber although no statistically significant difference was observed in the CPP difference score compared to Vehicle-Saline group (Vehicle + Saline vs. MJN110 + Saline: *p* = 0.64; CPA (<50°s): 45.45 vs. 61.54%) (Figures 5B,C).

CB2R is another member of the endocannabinoid system we found to be regulated by repeated morphine treatment. Previously, the activation of CB1R has been repeatedly demonstrated to promote opioid-induced reward (Chaperon et al., 1998; Navarro et al., 2001; Caillé and Parsons, 2003, 2006; De Vries et al., 2003; Solinas et al., 2003; Singh et al., 2004; Rashidy-Pour et al., 2013; He et al., 2019). In the present study, we sought to investigate if CB2R can modulate sustained morphine-induced rewarding behaviors. We pre-treated the rats with a selective CB2R agonist, JWH015 (3 mg/kg, i. p.), prior to morphine or saline treatment. The results showed that the activation of CB2Rs markedly inhibited morphine-induced preference (Vehicle + Morphine vs. JWH015 + Morphine: *p* < 0.05; CPP (>50°s): 88.89 vs. 56.25%) (Figures 5B,C), while JWH015 treatment alone did not present preference to any chamber (Figures 5B,C).

## Discussion

The ECS has emerged as a hot topic in the study of opioid reward, given the large body of evidence linking this system to the formation and development of opioid reward, withdrawal and addiction (Chaperon et al., 1998; Ledent et al., 1999; Navarro et al., 2001; Caillé and Parsons, 2003, 2006; De Vries et al., 2003; Solinas et al., 2003; Singh et al., 2004; Solinas et al., 2005; Luchicchi et al., 2010; Rashidy-Pour et al., 2013; Grenald et al., 2017; He et al., 2019). Recent studies showing promising synergistic effects of cannabinoids and opioids in chronic pain treatment further stimulate the interest of using cannabinoids to treat opioid abuse potential (Cichewicz, 2004; Tham et al., 2005; Bushlin et al., 2010; Abrams et al., 2011; Kazantzis et al., 2016; Grenald et al., 2017). However, very few studies have investigated the possible changes of ECS in the VTA - a key brain region mediating reward, in the presence of opioids, and no study has provided a comprehensive proteomic analysis of VTA following repeated opioid administration.

In the present studies using unbiased global proteomic analysis, we identified the expression of 31 proteins that belong to five different categories of the endogenous cannabinoid system in the VTA. Two proteins, PLCγ2, and ABHD6, were significantly downregulated by repeated morphine treatment. PLCγ2 is an enzyme belonging to the phospholipase C family that selectively hydrolyzes phosphatidylinositol 4, 5-biphosphate and generates diacylglycerol (DAG) (Kadamur and Ross, 2013). As DAG is the precursor of 2-AG, this downregulation of PLCγ2 may directly reduce the levels of 2-AG in the VTA. It is also possible that this decreased PLCγ2 expression may be a mechanism by which opioids modulate growth factor receptor-mediated synaptic regulation. Indeed, PLCγ2 is primarily activated by tyrosine protein kinases, such as growth factor receptors (Kadamur and Ross, 2013). ABHD6 is a newly identified member of the endocannabinoid system and contributes to approximate 4% of 2-AG hydrolysis measured in the whole mouse brain (Blankman et al., 2007), yet the ABHD6 activity is even higher than MAGL in select brain regions including select areas of the cortex, hippocampus, striatum and cerebellum (Baggelaar et al., 2017). Previous evidence suggests that ABHD6 controls the long-term synaptic depression in the central nervous system mediated via the 2-AG/CB1R axis or an endocannabinoid-independent AMPA receptor pathway (Marrs et al., 2010; Wei et al., 2016; Cao et al., 2019). The downregulation of ABHD6 expression following sustained morphine exposure may be a mechanism underlying opioid-mediated long-term synaptic depression and participate in opioid reward. Interestingly, our qPCR result showed that the expression of ABHD6 was not altered at the mRNA level, suggesting its downregulation detected by the proteomics is not at the transcription level. CB2R is a critical member of the endogenous cannabinoid system but is barely detected by current mass spectrometry-based techniques due to its low expression level (Marchalant et al., 2014). Our qRT-PCR experiments found that CB2R was significantly decreased by repeated morphine treatment. This downregulation of CB2R may be involved in a regulatory process of sustained morphine in the facilitation of the rewarding behavior (Xi et al., 2011; Navarrete et al., 2013; Ortega-Álvaro et al., 2015; Grenald et al., 2017). Overall, these studies suggest that sustained opioids exert regulatory effects on the VTA endogenous cannabinoid system.

Interestingly, our proteomic analysis indicates the endopeptidase inhibitors, particularly serine-type endopeptidase inhibitors, including α-1-macroglobulin, was the most significantly affected protein due to repeated morphine with over a 9-fold decrease. Considering the essential roles of these endopeptidase inhibitors in the regulation of synaptic plasticity and inflammation (Falkenberg et al., 1995; Almonte and Sweatt, 2011; Wang and Sama, 2012; Wake et al., 2016; Krause et al., 2019; Zhang et al., 2020), the modulation of these proteins by repeated morphine treatment may implicate novel regulatory mechanisms of opioids in the mediation of reward and addiction. It is worth mentioning that 18 out of the 45 upregulated proteins in our proteomic analysis were found to possess cleavage sites recognized by serine-type endopeptidases (Predicted by a peptidase database MEROPS (Rawlings et al., 2018); (Supplementary Table S4), suggesting the upregulation of these proteins may be attributed to the reduction of those endopeptidase inhibitors. As some of these upregulated proteins participate in the regulation of protein translation (Supplementary Table S4) this may indicate a novel pathway that opioids control different biological processes.

We also examined the modulatory effects of sustained morphine on the VTA levels of endocannabinoids, 2-AG and AEA. Our results exhibited that sustained morphine did not alter the production of either 2-AG or AEA in the VTA. These results are consistent with the unaltered protein expression of the major regulators of endocannabinoid production or degradation, such as DAGLα, MAGL, NAPE-PLD, and FAAH, and this lack of alteration is not affected by the time of the last morphine injection. In contrast, previous studies showed that sustained opioid changes the production of these endocannabinoids in several brain regions associated with reward process (i.e., striatum) although the extent and direction may vary (González et al., 2003; Viganò et al., 2003; Viganò et al., 2004; Caillé et al., 2007). Currently, we do not know the actual reasons why endocannabinoids are regulated differently among distinct brain regions, yet previous studies show that the changes of endocannabinoid production are not universal in all tested brain regions (González et al., 2003; Viganò et al., 2003; Viganò et al., 2004). Furthermore, the pattern of the drug administration may also play a role here as a substantial difference of the neurochemical, proteomic and genomic effects were observed when drugs were applied noncontingently or by free-choice self-administration (Jacobs et al., 2003). When sustained morphine was applied via daily injection (non-contingent), the AEA level in the NAc shell was not altered while self-administration (contingent) of heroin significantly increased AEA production (Viganò et al., 2004; Caillé et al., 2007). Lastly, different dosages and duration of opioid treatment may also influence the results. Interestingly, our study found that a single dose of morphine just prior to VTA-microdialysis collection significantly reduced AEA production in both sustained morphine- and saline-treated rats, suggesting that opioids may induce an acute regulation of AEA production in the VTA. The actual significance for this phenomenon is unclear and no other study has reported this result. Considering the relative higher affinity of AEA to CB1Rs than to CB2Rs as well as the facilitatory effect of CB1Rs on opioid-induced CPP, this reduction of AEA after morphine treatment may reflect a negative feedback mechanism in opioid reward. However, it is also possible that this reduction of AEA could be a result of handling stress on animals and/or a depletion of AEA after a 240 min period of collection. This did not occur for 2-AG levels suggesting less evidence for stress handling of animals, yet the regulation of the endogenous cannabinoids may be under different stress controls and cannot be ruled out. Further experiments analyzing the AEA alteration in acute saline-treated rats will address this question.

Although 2-AG does not seem to be modulated by sustained morphine, we found that modulating 2-AG production can regulate sustained morphine-induced reward. Our study identified that enhancing systemic 2-AG tone by a selective MAGL inhibitor, MJN110, significantly attenuated morphine-induced preference, suggesting an inhibitory effect of 2-AG on the rewarding behavior of morphine. Interestingly, we also found that activation of CB2Rs with a selective CB2R agonist, JWH015, significantly decreased sustained morphine-induced reward, suggestive of an opposite role of CB2Rs in opioid reward compared to CB1Rs. This result also suggests that the inhibitory effect of 2-AG on opioid reward is possibly mediated via CB2R rather than CB1R that promotes the opioid-induced rewarding behaviors. The functional difference of CB1R and CB2R in opioid reward may be caused by their distinct localization in the VTA. Previous studies found that CB1Rs are abundantly expressed on synaptic terminals targeting dopaminergic neurons in the VTA, indicating that CB1Rs serve as an autoreceptor and disinhibit GABAergic suppression of dopaminergic neuron activity (Fitzgerald et al., 2012; Van Bockstaele, 2012; Rashidy-Pour et al., 2013). In contrast, CB2Rs are primarily located on postsynaptic dopaminergic neurons, suggesting an inhibitory role of this receptor in reward process. Although no study has directly demonstrated this idea in opioid reward, this seems to be true in cocaine-induced reward (Zhang et al., 2014; Zhang et al., 2017). Further studies that directly modulating the CB2Rs expressed on the VTA dopaminergic neurons will provide the answer.

The endogenous cannabinoid and opioid systems are two critical neuromodulatory systems, which share similar pharmacological features, including the downstream signaling of µ-opioid receptors (MORs) and CB1Rs and behavioral outcomes (analgesia, sedation and reward) (Wenzel and Cheer, 2018). Recent evidence shows that these two systems are functionally interacted in the modulation of reward and addiction. Δ9-Tetrahydrocannabinol (THC)-induced CPP is eliminated in MOR-knockout mice (Ghozland et al., 2002) and THC self-administration is attenuated by opioid receptor antagonist naloxone (Braida et al., 2001; Justinova et al., 2004). Reciprocally, genetic depletion of CB1Rs or the application of CB1R antagonist rimonabant blocks opioid-induced CPP and self-administration (Ledent et al., 1999; Martin et al., 2000; Cossu et al., 2001; Navarro et al., 2001; Navarro et al., 2004; Caillé and Parsons, 2006). Currently, the underlying mechanisms of the functional interactions between the two systems remains to be elucidated, but the interactions between MOR and CB1R may be an explanation (Wenzel and Cheer, 2018). Indeed, these two receptors are both expressed on the GABAergic terminals in the VTA (Mátyás et al., 2008; Kudo et al., 2014), and the blockade of either receptor significantly decreases drug-induced dopamine release in the NAc (Chen et al., 1990; Tanda et al., 1997; Mascia et al., 1999). Importantly, Schoffelmeer et al. reported that MOR and CB1R may form heterodimers in the NAc and can cause synergistic suppression of GABA release, providing the possibility that the MOR and CB1R in the VTA may follow a similar pattern although additional *in vivo* studies are necessary to demonstrate if this is the case in the VTA (Schoffelmeer et al., 2006). Our present study provided evidence suggesting additional components of the endocannabinoid system may also involve in the functional interactions between the endogenous cannabinoid and opioid systems. However, more specific data using genetically modified animals are required to confirm this thought.

Overall, our current study provides a better picture of the ECS-related proteins expressed in the VTA and identified the expression of several proteins/genes (i.e., PLCγ2, ABHD6, and CB2R) were reduced after systemic sustained morphine exposure. We also evaluated the VTA levels of 2-AG and AEA; finding that AEA was reduced after acute exposure to morphine. These studies, for the first time, offer a comprehensive picture of the alterations of the VTA endocannabinoid system following sustained morphine exposure in male rats, providing several uncharacterized targets that may play a role in the regulation of opioid reward and addiction. Similar studies are ongoing in females to determine whether there are sex differences in the ECS after repeated morphine. It is also necessary to mention that systemic administration of morphine was performed in this study to mimic the route of administration and repeated use of opioids in humans (Ballantyne and Mao, 2003; Surratt et al., 2011; Volkow and McLellan, 2016) with the understanding that alterations in the ECS in the VTA may be direct or indirect. Lastly, our study identified the possibility that broad manipulation of the endocannabinoid system may mitigate opioid abuse potential directly addressing the ongoing opioid epidemic.

## Data Availability

The mass spectrometry proteomics data have been deposited to the ProteomeXchange Consortium via the PRIDE partner repository with the dataset identifier PXD023096 and 10.6019/PXD023096.

## Abbreviation

2-AG, 2-arachidonoylglycerol; ABHD6, α/β-hydrolase domain containing 6; AEA, anandamide; CB1R, cannabinoid receptor 1; CB2R, Cannabinoid receptor 2; CPP, conditioned place preference; DAGLα, diacylglycerol lipase; ECS, endogenous cannabinoid system; GO, Gene Ontology; MAGL, monoglyceride lipase; NAc, nucleus accumbens; PLCγ2, phospholipase Cgamma-2; VTA, ventral tegmental area.
